# Supplementary material for: Electromigration of Charged Analytes Through Immiscible Fluids in Multiphasic Electrophoresis
Source: Electrophoresis. 2024 Dec 2;46(1-2):13–21. doi: 10.1002/elps.202400192 (PMC11773303; doi:10.1002/elps.202400192)
Supplement: Supplementary file 1 — Supporting Information [file ELPS-46--s001.docx]

**Supplementary Information**

**Experimental Workflow:**

**
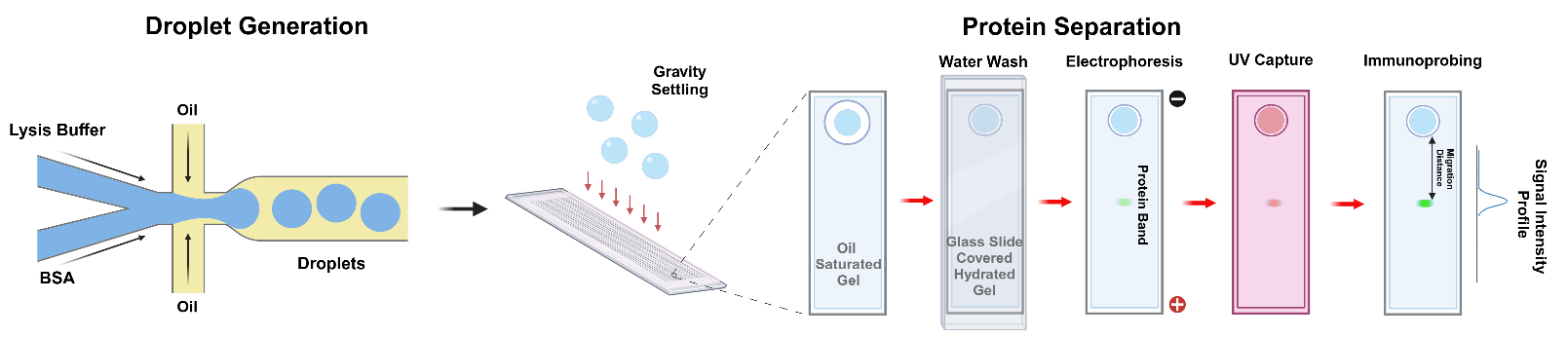
**

Figure S1: Experimental workflow for droplet settling electrophoresis and migration distance measurement. This figure was created with BioRender.com.

**Governing Equations for COMSOL Simulation:**

1. Electric Current Module

Ohm’s Law:

𝐉 = σ𝐄

where:

- 𝐉 is the current density,
- σ is the electrical conductivity,
- 𝐄 is the electric field, 𝐄 = −∇φ.

Gauss’s Law:

∇ · 𝐉 = 𝑄𝑗

where:

- 𝑄𝑗 is the current source (if any).

Resulting Poisson's Equation for Electric Potential:

∇ · (σ∇φ) = 𝑄𝑗

In regions with no current sources, this simplifies to:

∇ · (σ∇φ) = 0

2. Transport of Dilute Species Module

Nernst-Planck Equation:

∂cᵢ/∂t + ∇ · 𝐍ᵢ = 𝑅ᵢ

where:

- cᵢ is the concentration of species ᵢ,
- 𝐍ᵢ is the flux of species ᵢ,
- 𝑅ᵢ is a reaction term (if any).

Flux Term (Nernst-Planck Equation):

𝐍ᵢ = −Dᵢ∇cᵢ − zᵢμᵢcᵢ∇φ + 𝐮cᵢ

where:

- Dᵢ is the diffusion coefficient of species ᵢ,
- zᵢ is the charge number of species ᵢ,
- μᵢ is the mobility of species ᵢ,
- 𝐮 is the velocity field (if coupled with a fluid flow model).

**Boundary Conditions:**

Electric Currents Module:

- Electric Potential: φ = φ₀ (at electrode boundaries)
- Electric Insulation: 𝐧 · (σ∇φ) = 0

Transport of Diluted Species Module:

- Concentration Boundary Condition: cᵢ = cᵢ₀
- No-Flux Boundary Condition: 𝐧 · 𝐍ᵢ = 0

**Non-dimensionalization Approach Inspired by Probstein's Methodology**

We introduce *L'* and *w* as the axial and radial dispersion characteristic length scales, respectively, with *w* being the microwell's radius and *L'* derived from *w* as follows ^1^:

$L'=2\left( \frac{\mu_{ii}}{\mu_{i}} \right)w$….(S1)

Where *µ_ii_* and *µ_i_* are the electrophoretic mobilities of BSA in the two mediums present at the interface. For example, in case of scPAGE medium ii will be gel and medium i will be free solution whereas in case of DropBlot medium i will be oil. We calculate the diffusivity and electrophoretic mobility of BSA in mineral oil using Stokes Einstein equation (mineral oil viscosity: 0.1 Pa.s and BSA hydrodynamic radius: 3.9 nm). For the electrophoretic mobilities in PAGE gel and free solution, we use values that has been reported previously (µ_gel_: 5.25x10^-9^ m^2^V^-1^s^-1^ and µ_free solution_: 1.58x10^-8^ m^2^V^-1^s^-1^) ^2^. Using these characteristic lengths, we can compare the time scale of diffusion (*t_axial_ = L*'*^2^/D_ii_* and *t_radial_ = w^2^/D_ii_*), with the timescale for electrophoretic injection (*t_injection_ = L/v*). Where *D_i_*_i_ and *v* are the diffusion coefficient and the average velocity under an applied electric field of BSA in medium ii, respectively. We also define Peclet number (Pe) which compares the characteristic time for diffusion and advection as ^1^:

$Pe=\frac{L'}{L}\frac{wv}{D_{ii}}$….(S2)

Where *L* is the separation length. This definition allows us to analyze Pe for different separation lengths. The total dispersion itself is the sum of dispersions arising from injection, radial and axial diffusion:

$\sigma^{2}=\sigma_{inj}^{2}+\sigma_{radial}^{2}+\sigma_{axial}^{2}$ ….(S3)

Where, *σ_inj_^2^*, *σ_radial_^2^* and *σ_axial_^2^* represent the peak variance contributions from injection, radial, and axial diffusion, respectively. The temporal and spatial evolution of a sample peak post-injection can be modeled using Taylor-Aris dispersion. According to the Taylor-Aris dispersion model, the variances for a circular microwell geometry are derived as follows ^3–5^:

$\sigma_{inj}^{2}=0.25{(\frac{\mu_{ii}}{\mu_{i}})}^{2}w^{2}$….(S4)

$\sigma_{radial}^{2}=\frac{1}{X}(1-2\frac{\mu_{ii}}{\mu_{i}}){(w-\sqrt{w^{2}-8D_{ii}t})}^{2}$….(S5)

$\sigma_{axial}^{2}=2D_{ii}t$….(S6)

Here X is a constant determined by the shape of the input response function (IRF) which contributes to the excess variance. For a Gaussian IRF, X=16 ^1^.

**Electric Field Strength Across Oil Layer**

**
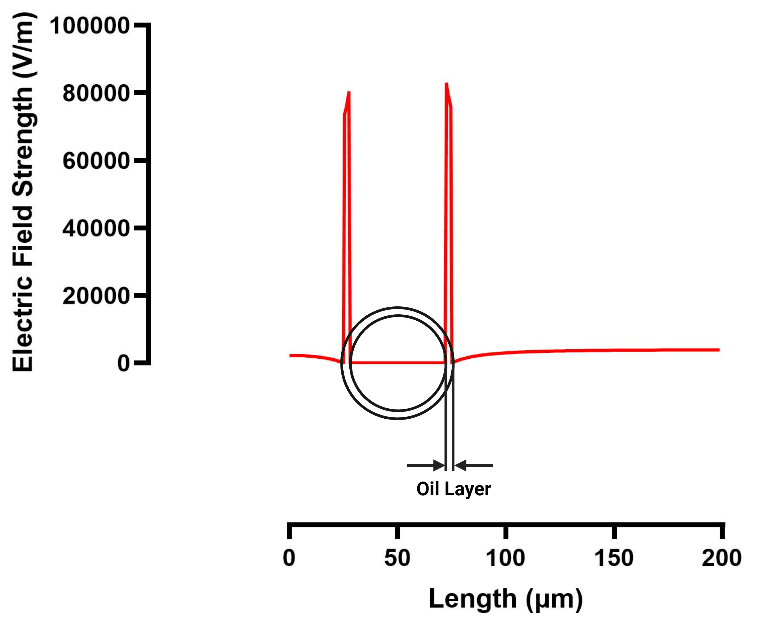
**

Figure S2: Simulation results of electric field strength across an aqueous droplet, through an oil layer, and into a polyacrylamide gel. The simulation demonstrates a sharp increase in electric field strength upon entering the oil layer, followed by a uniform distribution throughout the gel layer.

**Droplet Settling in an Elliptical Microwell**


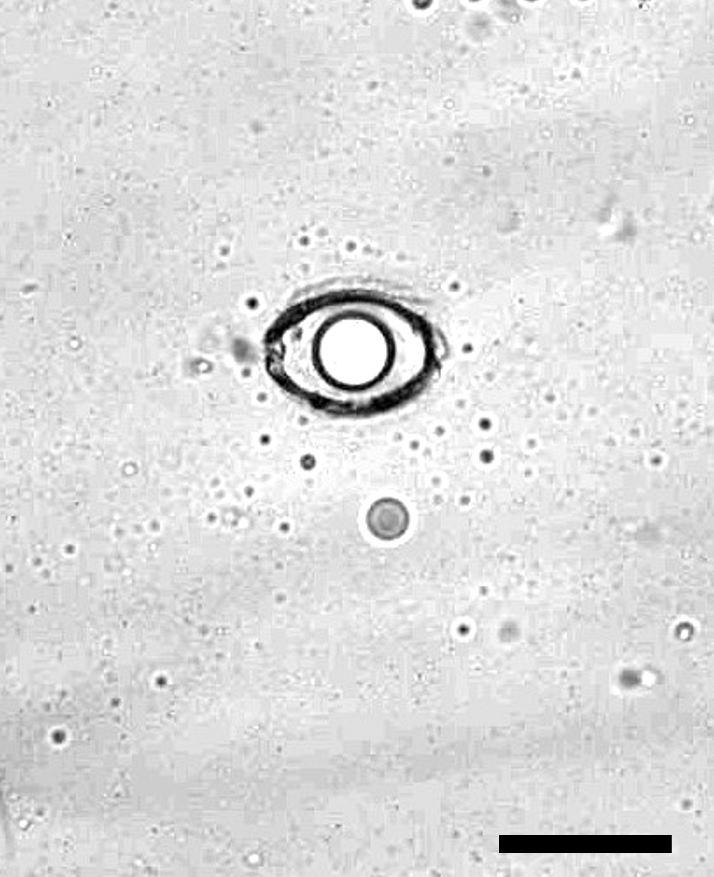


Figure S3: A droplet, gravity settled inside of an elliptical microwell. Scale Bar: 100 µm.

**Poisson Distribution of Droplet Settling into Microwells of Varying Eccentricities**

**
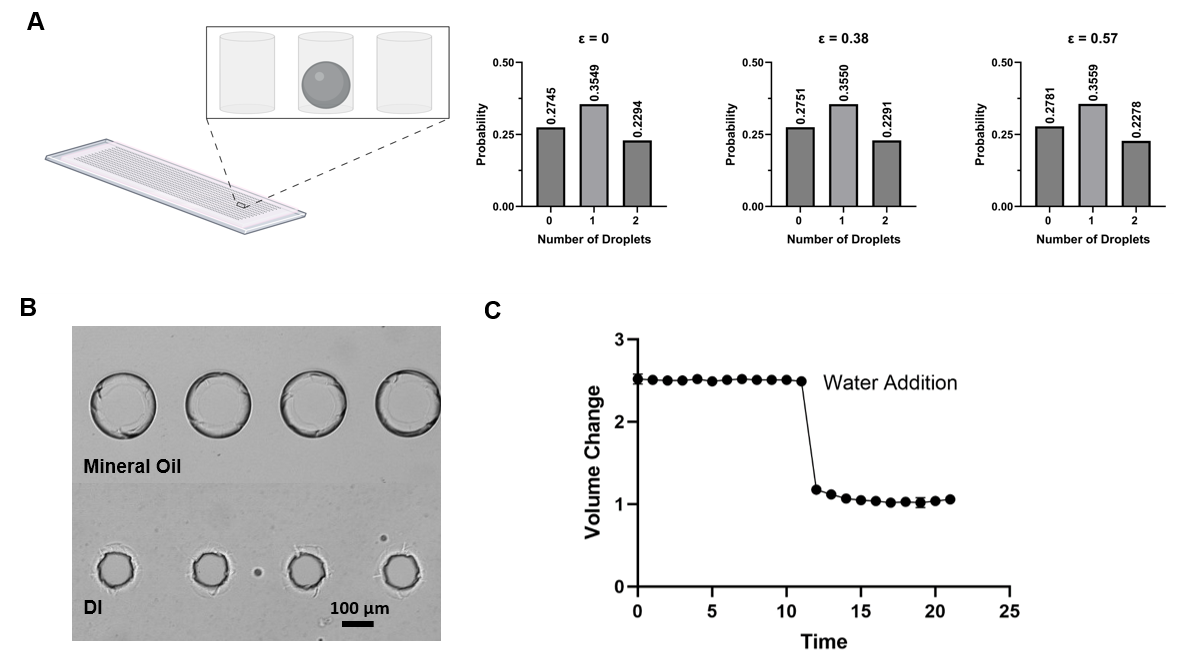
**

Figure S4: (A) Poisson distribution analysis of 0 1 and 2 droplet settling in microwells with different eccentricities. (B) Microwell expansion and contraction upon oil and water treatment. (C) Microwell hydration dynamics.

**Experimental Results for Multiple Electrophoresis Lanes**


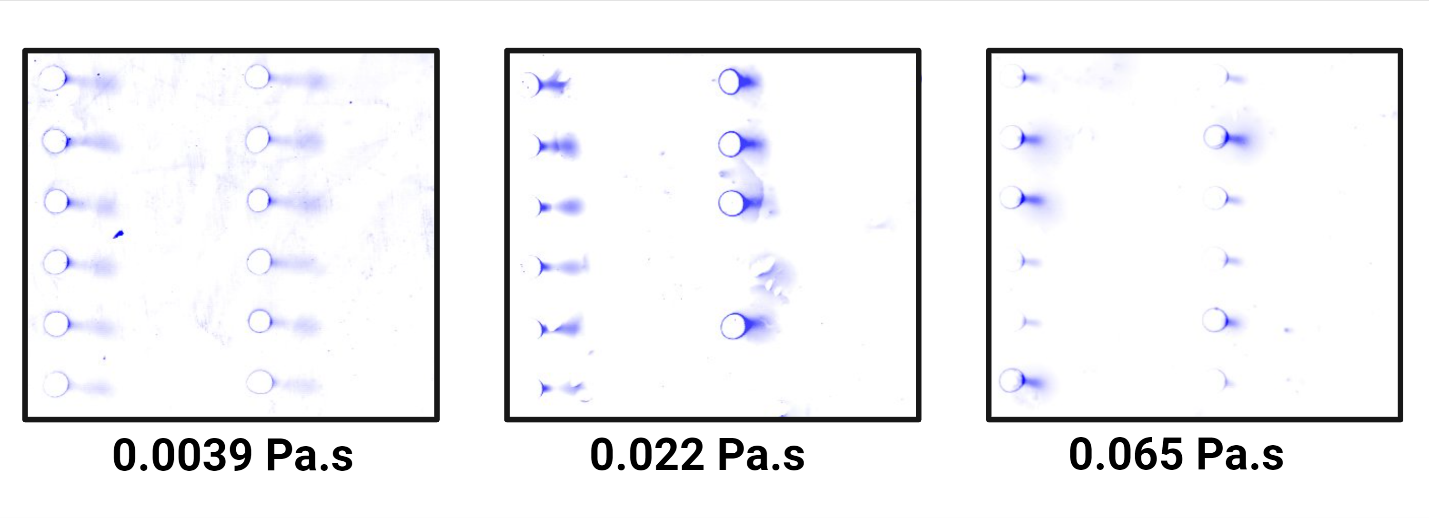


Figure S5: (A) Electro-injection of BSA from aqueous droplets through a thin oil layer into polyacrylamide gels.

**Reference**

(1) Pan, Q.;Herr, A. E. Geometry-Induced Injection Dispersion in Single-Cell Protein Electrophoresis. *Anal Chim Acta* **2018**, *1000*, 214–222. https://doi.org/10.1016/j.aca.2017.11.049.

(2) Grist, S. M. Mourdoukoutas, A. P. Herr, A. E. 3D Projection Electrophoresis for Single-Cell Immunoblotting. *Nat Commun* **2020**, *11* (1), 1–17. https://doi.org/10.1038/s41467-020-19738-1.

(3) Sternberg, J. C. *Advances in Chromatography*; Giddings, J. C., Keller, R. A., Eds.; New York, **1966**; Vol. 2.

(4) Taylor, G. I. Dispersion of Soluble Matter in Solvent Flowing Slowly through a Tube. *Proceedings of the royal society A* **1953**, *219* (1137).

(5) Aris, R. On the Dispersion of a Solute in a Fluid Flowing through a Tube. *Proceedings of the royal society A* **1956**, *235* (1200).
